# Tissue engineering modalities in skeletal muscles: focus on angiogenesis and immunomodulation properties

**DOI:** 10.1186/s13287-023-03310-x

**Published:** 2023-04-15

**Authors:** Atieh Rezaei Namjoo, Fateme Nazary Abrbekoh, Sepideh Saghati, Hassan Amini, Mohammad Ali Ebrahimi Saadatlou, Reza Rahbarghazi

**Affiliations:** 1 Stem Cell Research Center, Tabriz University of Medical Sciences, Tabriz, Iran; 2 Student Research Committee, Tabriz University of Medical Sciences, Tabriz, Iran; 3 Department of Tissue Engineering, Faculty of Advanced Medical Sciences, Tabriz University of Medical Sciences, Tabriz, Iran; 4 General and Vascular Surgery Department, Tabriz University of Medical Sciences, Tabriz, Iran; 5 Department of Basic Sciences, College of Veterinary Medicine, Tabriz Branch, Islamic Azad University, Tabriz, Iran; 6 Department of Applied Cell Sciences, Faculty of Advanced Medical Sciences, Tabriz University of Medical Sciences, Tabriz, Iran

**Keywords:** Muscle tissue regeneration, Engineering approaches, Hydrogels, Angiogenesis, Immunomodulation

## Abstract

Muscular diseases and injuries are challenging issues in human medicine, resulting in physical disability. The advent of tissue engineering approaches has paved the way for the restoration and regeneration of injured muscle tissues along with available conventional therapies. Despite recent advances in the fabrication, synthesis, and application of hydrogels in terms of muscle tissue, there is a long way to find appropriate hydrogel types in patients with congenital and/or acquired musculoskeletal injuries. Regarding specific muscular tissue microenvironments, the applied hydrogels should provide a suitable platform for the activation of endogenous reparative mechanisms and concurrently deliver transplanting cells and therapeutics into the injured sites. Here, we aimed to highlight recent advances in muscle tissue engineering with a focus on recent strategies related to the regulation of vascularization and immune system response at the site of injury.

## Introduction

Skeletal muscles are consolidated tissue and consist of aligned multinucleated myocytes, satellite cells, nerves, blood vessels, and extracellular matrix (ECM) in complex structures [1–3]. Like other tissues, skeletal muscles are sensitive to varied acute and chronic injuries caused by physical trauma, plastic/cosmetic surgeries, arterial occlusion, metabolic diseases, peripheral nerve atrophies, congenital diseases, etc. [4–12]. Following an injury, the promotion of inflammatory response leads to the activation of the quiescent skeletal muscle stem cells (known also satellite cells) for the regeneration of injured sites via proliferation, differentiation, and subsequent cell-to-cell fusion [8, 13, 14]. Despite the existence of a sophisticated healing mechanism within the parenchyma of muscular tissue, a high proportion of satellite cells are damaged when the injury is extensive and the basal lamina is disrupted. Under such conditions, chronic inflammation and dysregulated macrophage response can lead to inappropriate regeneration, excessive collagen fiber deposition, and fibrotic changes [15]. Along with these changes, abnormal ECM synthesis via recalled fibroblasts dominates myogenesis, leading to the formation of non-functional scar tissue [16, 17]. Scar tissue can deteriorate the integrity of the capillary and neural network and results in the loss of suitable vascularization and denervation. These features affect the basal metabolism of residual tissue and promote muscle atrophy and loss [15, 18, 19]. In line with these facts, therapeutic interventions should be eligible to prevent or minimize unwanted inflammatory consequences and improve the regeneration rate. In recent decades, muscle flap transfer from donor sites to the injured tissues is touted as the gold standard in clinical therapy following muscle damage/volumetric muscle loss (VML). Although autograft transplantation can orient vascular and neural networks to the injury site this approach is limited due to donor site availability, morbidity, and poor engraftment rate [20–22]. Besides, the application of cadaveric allografts is also hindered by the lack of sufficient organ donors and the risk of disease transmission [22–24]. Moreover, the diversity of defects in shape and location in different cases and complications such as necrosis or post-surgery infections make the surgery a challenging procedure that requires professional and experienced surgery teams [25–27]. In cell-based modalities, the application of myogenic or non-myogenic cells is also restricted due to the low viable cell rate during transplantation into the injured sites. To overcome these limitations, protective microenvironments with proper biochemical and mechanical cues are essential to dictate specific cellular behavior and function [28, 29]. In recent years, tissue engineering, a branch of interdisciplinary science, can help researchers and clinicians with accelerated tissue healing using de novo technological approaches consisting of specific cell populations, growth factors, and scaffolds [30, 31]. In this article, recent data associated with the application of varied scaffold types for the regeneration of injured muscle tissue was investigated.

## Application of natural hydrogels for muscle regeneration

Hydrogels possess 3D hydrophilic polymer networks with the ability to maintain a large amount of aqueous phase without getting dissolved [32, 33]. Based on the components and materials used, hydrogels are classified into three distinct categories as follows; natural, synthetic, and hybrid hydrogels with several advantages and disadvantages (Table 1) [34]. It was indicated that natural hydrogels have physical properties similar to the native ECM (Table 2) [35]. Because of injectability and flexible structure, hydrogels can adapt themselves appropriately to the geometry of irregular injuries and wounds [36]. The exchange of substances in the liquid phase makes hydrogels suitable substrates for drug and cell delivery to the target sites with minimum invasion [37]. It has been suggested that hydrogels are suitable candidates for the engineering of injured skeletal muscles as described for other tissues [38–41].

**Table 1:** Advantages and disadvantages of hydrogels based on their source

Hydrogel type	Advantages	Disadvantages	Refs.
Natural hydrogels	Bioactive and friendly microenvironments for encapsulated cells in in vivo and in vitro conditions Several anchor spots and binding sites can promote cell attachment, morphological adaptation, and cytoskeletal organization Lack of excessive immune system responses because of their natural sources Induction of survival, migration, and differentiation of cells Appropriate ECM modeling Induction of several signaling pathways Angiogenesis/Vascularization	Poor physical characteristics and mechanical stability Difficult handleability and manipulation High-priced and time-consuming synthesis and sterilization protocols Rapid degradability Non-functional fibrosis formation	[64, 302–307]
Synthetic Hydrogels	Economical and low-cost synthesis protocols Relatively rapid and easy synthesis protocols Appropriate for large-scale utilization High mechanical characteristics, Suitable handleability, and tunability Appropriate for advanced therapeutic applications	Lack of appropriate adhesion sites and bioactive molecules leading to the interposition of the regeneration process Reduction of cellular functionality Possibility of foreign body responses due to their oil-based sources or toxic secondly substance	[308–310]
Hybrid Hydrogels	Diversity in starting materials and components Extensive usage in regeneration applications Advantages depend on the material combination	High-priced and time-consuming synthesis protocols Disadvantages depend on the material combination	[311, 312]

**Table 2:** Natural substrates used for muscle tissue regeneration

Study	Hydrogel type	Outcomes	Ref
Rat and mouse models of hind limb ischemia	Decellularized skeletal muscle ECM	Restoration of blood perfusion, Induction of angiogenesis, and ECM concentration affect the viscosity, physical strength, and hydrogel degradation rates	[313]
Mouse model of acute right tibialis anterior muscle injury	Decellularized ECM hydrogel	Increase in pax 7, Upregulation of nnt3, Tcap, Jsrp1, Mylk2 in tissue-specific ECM hydrogel	[314]
In vitro culture of Lewis rats satellite cell	ECM component and plant-derived component hydrogel	Fibrin hydrogel determined as the most qualified scaffold for satellite cell culture and skeletal muscle regeneration	[315]
In vitro & in vivo: diaphragm defect of BALB/c Rag2^−/−^ mice	Decellularized ECM of diaphragm tissue of piglet	This hydrogel was determined as a favorable acellular scaffold for diaphragm injuries	[316]
In vitro	Transglutaminase cross-linked gelatin hydrogels	This hydrogel increases the myotube length on isometric gelatin hydrogel with low stiffness. Long-term cell culture induced contractile phenotype and upregulates MHC	[317]
In vitro & in vivo: hind limb ischemia of mouse model	RGD-modified D-form peptide hydrogel (Nap ^D^F^D^FKGRGD) with mesenchymal stem cells	Hydrogel demonstrated favorable biocompatibility and stability after implantation on hind limb ischemia of mice with enhanced cell survival and pro-angiogenesis properties	[318]
In vitro	Decellularized ECM of bovine pericardium hydrogel	Hydrogel-supported C2C12 viability, upregulation of MHC, Myogenin, and α-SMA	[319]

ECM is composed of highly organized proteins, proteoglycans, and glycoproteins in macro and microstructures to provide physicochemical cues for cell function and bioactivity [42, 43]. It is thought that the composition of ECM differs from tissue to tissue. Certain heterogeneous networks of ECM provide a stable 3D platform with the capacity to support mechanical forces, and modulate external pressure, especially in load-bearing tissues such as bone and muscles. Besides, ECM acts as the reservoir of growth factors and varied signaling molecules to dictate specific cell function by providing biochemical cues for reciprocal cell-to-cell and cell-to-ECM interactions [43, 44]. These features help cells to maintain their homeostasis via the regulation of proliferation, differentiation, and migration within the surrounding matrix [45, 46]. Due to proper biomimetic microenvironments, decellularized ECM is considered one of the most promising scaffolds/hydrogel types in preclinical and clinical tissue engineering applications [47, 48].

Despite their advantages in the promotion of tissue healing, it should be considered that decellularization methods and ECM sources influence the physical characteristics, permeability, and degradation rate of the final product [49–51]. To date, different tissue sources have been utilized to create ECM hydrogels for the regeneration of muscular tissue such as the urinary bladder and small intestines [52, 53], but the application of skeletal muscle ECM is the optimal source. Molecular investigations have revealed that skeletal muscle ECM possesses composition and niche close to native tissue even after the decellularization process. For instance, certain ECM components such as laminin *α*_1_ and *α*_2_ are abundant in the decellularized skeletal muscle ECM [54, 55]. This strategy can accelerate the regeneration process in injured muscles in a similar way that occurs in native tissues under pathological conditions.

In this context, Ungerleider et al. investigated the effect of muscle-ECM hydrogel on the regeneration of muscle tissue in comparison to lung-ECM hydrogel in a mouse model [56]. Both decellularized ECM types were prepared using the same detergents. The authors demonstrated increased Pax7^+^ muscle progenitor cells and large-sized myofibrils in the right tibialis anterior muscles with tissue-matched ECM. These data show the superiority of tissue-specific ECM hydrogel in an efficient regeneration outcome [56]. Compared to allograft ECM hydrogels, xenogeneic tissue sources are other options to prepare ECM hydrogels. Despite their availability, the transmission possibility of infectious agents and inflammatory reactions should not be neglected. It is thought that the remnant of a Gal epitope or foreign DNA residue can promote the activity of immune system components after transplantation into the host tissues [57, 58].

Along with the application of an acellular matrix to enhance the regeneration rate in muscular tissue, some authorities have applied ECM components individually or in combined form in the final hydrogel composition. Notably, collagen, fibrin, keratin, gelatin, or non-mammalian sources like chitosan, alginate, or silk (solo or in different combinations) have been used for skeletal muscle regeneration [59–61].

Despite their regenerative potential, mechanical properties and biophysical features are the most challenging issues in the hydrogel form [62, 63]. For instance, in a study, myogenic and rheological properties of different hydrogel types (type I collagen, agarose, alginate, fibrin, and collagen/chitosan) were compared. Data revealed the superiority of fibrin and collagen-based hydrogels in the promotion of the myogenic capacity of rat satellite cells in in vitro conditions [64]. Fourteen-day culture of satellite cells on these substrates increased significantly the expression of myogenesis key markers MyoD, Myogenin, and myosin heavy chain in the fibrin group related to other substrates [64]. Despite the myogenic properties and high extensibility, fibrin hydrogel was completely degraded after 5 days [64]. Further investigations are necessary to find the most suitable substrates with proper mechanical features and concurrent myogenesis capacity. Several attempts have been made to alter the structure of hydrogels using physical or chemical modalities to regulate the behavior of resident and transplanted cells and immune cell response [62, 63, 65–67]. To this end, hybrid hydrogels composed of synthetic and natural substrates are recent approaches in muscle tissue engineering (Table 3) [15–69]. These approaches enable us to sophisticatedly control physicochemical properties such as degradation and swelling rates.

**Table 3:** Hybrid hydrogel used in muscle tissue regeneration

Study	Hydrogel type	Outcomes	Ref
In vitro & in vivo: murine quadriceps as VML model	thiolated hyaluronic acid chondroitin sulfate-PEG hydrogel	Hydrogel-supported C2C12 myoblasts viability and growth and upregulates MyoD, MyoG, and MYH8. In vivo analysis indicates enhanced angiogenesis, myofibers formation, migration, and pax 7 expression in the injured area	[218]
In vitro & in vivo: tibialis anterior muscles of mice	methacrylic-acid (MAA)-collagen and MAA poly(ethylene glycol) hydrogels	(MAA)-Col hydrogel promotes thicker muscle fiber formation compared to PEG, Col, and control groups with simultaneous appropriate vascularization and innervation. Fibrosis and inflammation markers were reduced	[15]
In vitro	Ca-modified sodium alginate (SA)-polycaprolactone (PCL)-reduced graphene oxide nanohydrogel	Hybrid hydrogel demonstrated great electroconductivity without cytotoxicity. The adhesion and differentiation of mouse C2C12 myoblasts were induced	[110]
In vitro	Fibrin-tetraethoxysilane, Fibrin-aminopropyltriethoxysilane And fibrin-silica nanoparticles hydrogels	Fibrin hybrid hydrogel had higher mechanical properties and led to a higher C2C12 myoblast proliferation rate compared to pure fibrin hydrogel	[98]
In vitro	Polymerized calcium phosphate‒polyvinyl alcohol‒sodium alginate hybrid hydrogel	Hydrogel showed self-healing ability with elevated energy dispersal, mechanical stability, and high fracture point comparable/close to skeletal muscle tissue	[69]
In vitro	Nanocellulose-graphene oxide/poly[acrylamide-*co*-(acrylic acid)	Hybrid hydrogel showed repetitive self-healing ability with excellent tensile strength and high fracture point	[106]
In vitro & in vivo: female C57BL/6 J mice skin injury	Methacrylated gelatin-acryloyl-(polyethylene glycol)-N-hydroxysuccinimide ester-modified elastin	Appropriate flexible physiochemical properties were obtained by altering the elastin ratio. In vivo experiment indicated infiltration of neutrophils and M2 macrophages, resulting in enhanced angiogenesis	[320]

In recent years, plant-based hydrogels are emerging scaffolds over recent years [70–72]. Phytocompounds such as polysaccharides (agar, cellulose, and pectin) and proteins (soy and zein) are abundant in plants [73–76]. It has been shown that plant-based hydrogels are eco-friendly, low-cost, and biocompatible with low-rate biodegradation [77–79]. In an experiment conducted by Mehrali and co-workers, the biocompatibility of hydrogel composed of ultraviolet cross-linked pectin-methacrylate with thiolated gelatin was studied on the viability of mouse myoblast C2C12 cell line [38]. Data confirmed that the application of pectin-based hydrogel can promote the dynamic growth of skeletal muscle progenitor cells in in vitro conditions. The lack of mammalian-specific degrading enzymes makes phyto-hydrogels suitable scaffolds for long-term regeneration processes in certain tissues like muscular tissue [80, 81]. However, plant-based hydrogels face some limitations and challenges [82, 83]. For example, preliminary modifications are necessary for obtaining a suitable microenvironment after transplantation into the target sites [82, 84]. Besides, the optimum composition should be defined in terms of certain tissues.

## Application of synthetic hydrogels for muscle regeneration

In several studies, pure synthetic substrates such as PEG [85], PU [86], PLA [87], and PVA have been used for the regeneration of skeletal muscles (Table 4). Despite some limitations associated with the application of pure synthetic hydrogels, these substrates are often inexpensive and manufacturing does not require complex processes [88, 89]. The existence of certain physicochemical properties makes the synthetic hydrogels to be easily adapted to the spatial and biophysical features of targeted tissues [90, 91]. Unlike natural hydrogels, synthetic hydrogels do not have suitable hydrophilicity and mutual cell-hydrogel interaction is less due to the lack of signaling cues and attachment sites [92, 93]. In this regard, synthetic strategies should be directed in a way to include cell attachment molecules such as Wnt11 [24] and other signaling biomolecules for proper morphological adaptation and cell-to-cell and cell-to-ECM interaction [94, 95].

**Table 4:** Synthetic hydrogels for muscle tissue regeneration

Study	Hydrogel type	Outcomes	Ref
In vitro & in vivo: Tibialis anterior muscle defect of rat	PVA-silicate ion-releasing hydrogel	Hydrogel degradation and ion-releasing rate are similar to regenerating muscle. Hydrogel-supported angiogenesis and myogenesis while diminishing oxidative stress effects	[65]
In vitro & in vivo: Tibialis anterior muscles of mice	Maleimide groups functionalized four-arm PEG hydrogel	Hydrogel increased the population of Pax 7 cells and the migration of injected mouse stem cells	[24]
In vitro & in vivo: Female C57BL/6 mice Hhind limb Iischemia	Poly(NIPAAm-co-NAS-co-HEMA-HB4-co-PAA-co-MAPEG) containing CTT	Synthetic hydrogel acts as an MMP‒2 regulator to inhibit ECM degradation while boosting angiogenesis in ischemic skeletal muscle	[321]
In vitro	Reduced & unreduced graphene oxide/polyacrylamide (GO/PAAm) hydrogel	Reduced GO/PAAm led to higher upregulation of MHC, MyoD, and myogenin. Electrical stimulation of reduced GO/PAAm hydrogel exerted a stronger impact on MHC, MyoD, and myogenin expression	[322]
In vitro	Poly(ethylene glycol) diacrylate‒acrylic acid (AA) in the diverse component ratio	A 1:4 ratio of poly(ethylene glycol) diacrylate‒acrylic acid had the highest cell survival and metabolic activity	[323]
In vitro & in vivo: Ttibialis anterior muscle injury of rat	F-127‒AuNPs and F-127‒Au-AuNPs synthetic hydrogel	Hydrogels had different cytotoxicity rates. The upregulation of MyoD, MyoG, and Tnnt-1 was observed in both groups. Higher myofiber density was observed in the animal model	[324]
In vitro	Poly(N-isopropylacrylamide-co-2-hydroxyethyl methacrylate) with a diverse ratio of components	This hydrogel supports the adhesion, viability, and proliferation of C2C12 myoblasts	[325]

Elasticity is an essential critical factor in the fabrication of hydrogels in terms of skeletal muscle regeneration [64]. On this basis, Xu and colleagues previously investigated the impact of varied elastic moduli on myogenic differentiation of encapsulated rat bone marrow mesenchymal stem cells (BMMSCs) after 2 weeks within the synthetic hydrogel composed of acrylic acid, 2- hydroxyethyl methacrylate oligoester, and N-isopropyl acrylamide via the alteration of oligomer length [96]. Data revealed the maximum myogenic differentiation outcomes in rat BMMSCs after being exposed to 20 kPa moduli, whereas an elastic modulus of 40 kPa can increase the proliferation rate. These data confirm the impact of elasticity at different values on the dynamic activity of transplanted stem cells. In another study, the cytocompatibility of cross-linked PEGDA and acrylic acid hydrogel with different polymer concentrations was examined on mouse C2C12 myoblasts over a period of 10 days in the laboratory setting [97]. According to obtained data, maximum ECM synthesis, cell adhesion, and metabolic activity are achieved when the ratio of acrylic acid to PEGDA in final composites becomes 4:1, respectively.

Of note, excessive foreign body reactions by local macrophages and massive collagen fiber deposition are the main challenges associated with the application of pure synthetic hydrogel in terms of muscle regeneration [98, 99]. One effective strategy would be the inclusion of immunomodulatory factors in the backbone of synthetic hydrogel [100]. For example, the application of nanofibrous PCL/PLGA scaffolds loaded with sphingosine 1-phosphate receptor-3 antagonist, namely VPC01091, in mice with spinotrapezius muscle volumetric injury led to increased muscle progenitor cells activity, phenotype shifting of macrophages toward CD206^+^ M2 type, and reduction of immune cells in the target sites [101]. These features coincided with the reduced number of recruited CD4^+^ and CD8^+^ lymphocytes and bridging rate in newly generated myofibrils [101].

The promotion of angiogenesis is touted as a strategic approach for accelerating healing procedures in the injured area [102]. To avoid necrotic changes in transplant cells, supplementation of vascular beds to the implants seems critical [34]. In an experiment, the application of Fingolimod (FTY720) as a specificity protein 1 (Sp1) agonist can contribute to the stimulation of angiogenesis and reduction of fibrotic changes [103].

Along with the application of several signaling biomolecules in the structure of final composites to accelerate healing procedures, metal nanoparticles (NPs) have been widely used to increase the biochemical activity of hydrogels [104, 105]. Ge and colleagues studied the myogenic effects of Pluronic® F-127 hydrogel enriched with Au and Au/Ag NPs on mouse C2C12 myoblasts. Pluronic® F-127 hydrogel possesses an amphiphilic nature and is composed of poly(ethylene oxide) (PEO) and poly(propylene oxide) (PPO). Data indicated that Au-, Au/Ag-loaded Pluronic® F-127 hydrogel increased the expression of certain genes such as MyoD, MyoG, and Tnt-1 in in vitro conditions [106]. Injection of Au/Ag-loaded Pluronic® F-127 hydrogel into defective tibialis anterior in a rat model led to the formation of myofibers juxtaposed to the vascular bed [106]. In addition to metal NPs, other nano-sized structures have been used for the fabrication of hydrogels with myogenic capacities [107]. Graphene oxide (GO) with certain physicochemical properties and abundant functional groups (carboxyl, epoxy, and hydroxyl) has been used for the preparation of varied hydrogels [40, 108, 109]. It was indicated that the culture of C2C12 myoblasts on GO-polyacrylamide hydrogel up-regulated the transcription of MyoD, MyoG, and α-myosin heavy chain after 7 days. The incorporation of GO nanosheets cross-linked via zinc into a sodium alginate polymeric network indicated proper charge carrier movement, and a high value of dielectric loss which is associated with conductivity [110]. The electroconductive polymeric network can provide biomimetic platforms for electrical communication between the myocytes and the regulation of the neuromuscular junction [110]. Direct evidence for the stimulatory effect of conducting substrates on the myogenic activity of C2C12 myoblasts was highlighted previously by Tang and co-workers [111]. Based on the data, the culture of C2C12 myoblasts on a substrate consisting of poly(3,4ethylenedioxythiophene)/poly(styrenesulfonate) (PEDOT/PSS) and dopamine-polymerized PCL scaffold led to enhanced proliferation rate at optimum concentrations [111].

The self-healing property is one of the most interesting strategies in tissue engineering of muscle tissue [112, 113]. In self-healing scaffolds, the polymer networks are reconstituted after the disintegration of the backbone of the polymer due to the existence of specific chemical bonding. Under these circumstances, the release of a specific factor can act as a trigger to renovate the 3D structure. Compared to natural hydrogels, synthetic polymers benefit from strong mechanical properties and suitable stretchability which are required for self-healing hydrogels applied for load-bearing tissues like skeletal muscles [112–116]. Guo and co-workers fabricated a self-healing conductive injectable hydrogel consisting of dextran-graft-aniline tetramer-graft-4-forms benzoic acid and N-carboxyethyl chitosan for monitoring the induction of myogenesis [112]. It is postulated that the existence of dynamic Schiff base bonds between formyl benzoic acid and amine groups of N-carboxyethyl chitosan is responsible for hydrogel's self-healing ability [112]. Data revealed the fabricated hydrogel possesses appropriate injectability and linear degradation pattern [112]. The encapsulated C2C12 myoblasts and endothelial cells (ECs) were distributed uniformly within the hydrogel without local aggregation. Of note, the cells showed proper migration capacity and paracrine activity to produce several myokines, making the above-mentioned hydrogel suitable for cell delivery approaches in terms of muscle regeneration [112]. In one study, self-healing PEG hydrogel was fabricated with the formation of hydrazone bonds between aldehyde and hydrazine functional groups [117]. This hydrogel exhibited specific viscoelastic and gel properties under physiological pH and temperatures. In vitro analysis revealed the formation of multinucleated myocytes by encapsulating C2C12 myoblasts within self-healing PEG hydrogel [117]. The culture of ECs within the self-healing and glucose-sensitive poly(PEG-diacrylate-dithiothreitol (PEGDA/DTT) hydrogel with hollow tubular form activated certain genes such as CD31, eNOS, and VEGFR after 3 days in culture medium [118]. ECs cultured within the unique tubular structure mimic the native vessel-like niche after 14 days. It is believed that this technique can be appropriately used in the regeneration of injured muscle tissue due to vasculogenic properties.

## Chemical and non-chemical cross-linking in scaffold synthesis

### Non-chemical modifications

Reactions within the polymeric network of hydrogels can be used to reduce or avert dissolving capacity [119]. These reactions can be tailored by using physical and chemical cross-linking techniques [119, 120]. Gelation, based on physical cross-linking, can be promoted via non-covalent interactions such as hydrogen and coordinating bonds, and ionic and van der Waals interaction [121–124]. Because of dynamic features, non-covalent interactions increase the self-healing capacity and injectability of hydrogels. Despite these advantages, in hydrogels fabricated by non-covalent interactions, physical integrity can be easily eliminated after being exposed to the biofluids [125, 126]. The main challenges associated with the application of physically cross-linked hydrogels include a lack of appropriate control over the gelation step, non-adjustable degradation, and porosity [127]. Under these circumstances, the application of chemical cross-linking can improve biocompatibility and biodegradation rate [128]. Alternatively, various approaches can be used for improving the structure of final composites. For instance, the application of two or more physical cross-linking methods can increase structural stability via synergistic effects [129]. The formation of hydrogen bonds between PAACA and PVA and subsequent crystallization via polyvinyl alcohol groups led to the fabrication of pH-sensitive, self-healing hydrogel with a compatible tensile strength at different temperatures [130]. The cold-drawing method is a kind of linkage density enhancement technique for the improvement in mechanical features [131]. It confirmed that the cold-drawing method can improve hydrogen bonds in PAA/PVA hydrogel in terms of quantity and intensity [132]. This substrate exhibited appropriate elastic modulus (100 MPa) and tensile strength (140 MPa) [132]. The formation of a multi-physical linkage is another way to enhance the mechanical properties of the final composites [129]. It is thought that this approach is useful enough to yield hydrogel with appropriate biocompatibility and self–healing after energy dissipation [133, 134]. In some circumstances, heavy metals are applied to increase the number of hydrogen bonds and to reduce the reversibility of physical linkages. However, the risk of toxicity should not be neglected [135–137].

### Chemical modification

Until now, techniques associated with the formation of covalent bridges in polymeric networks have been used for the fabrication of chemically cross-linked hydrogels [138, 139]. The chemical approaches include cross-linker association, radiation, enzyme association, and click chemistry [128, 140–142]. In the first three methods, classic covalent linkages are generated within the polymeric structure [143, 144]. Chemically cross-linked hydrogels are stable because of their non-flexible structures. Of course, the level of irreversibility is associated with the certain chemical initiators used in the fabrication steps [144, 145]. Most chemically cross-linked hydrogels have irreversible structures hence known also as permanent hydrogels [146, 147]. In the click chemistry method, dynamic covalent bonds are initiated under mild reaction conditions [69]. In contrast to other available approaches, click chemistry provides hydrogels with reversible structure and injectability features, making them eligible for transplantation into irregular injury sites [148].

#### Cross-linking with additional molecules

In this method, specific small molecules are applied to form covalent links between functional groups [149]. The formation of covalent bridges can contribute to the formation of relatively stable hydrogels with prolonged durability. Ultrastructural analyses have indicated tunable structure via the regulation of cross-linkers content and variants [150, 151]. To date, several cross-linker types such as epichlorohydrin, glyoxal glutaraldehyde, formaldehyde, and ethylene glycol diglycidyl ether (EDGE) have been used for the fabrication of chemically cross-linked hydrogels [152–156]. In vitro and in vivo analyses have shown various degrees of mutagenicity, calcification, and cytotoxicity after being implanted into the target tissues [157, 158]. To circumvent these issues, various researchers have suggested alternative materials [129]. For example, the application of phyto-cross-linkers (known as green cross-linkers) can increase hydrogel dynamic behavior via the regulation of angiogenesis, inflammation, and differentiation capacity in transplanted cells [159–162]. Among different green cross-linkers cinnamaldehyde, epigallocatechin gallate, and genipin isolated from cinnamon tree, green tea, and *Genipa americana* fruit are commonly used in tissue-engineered hydrogel synthesis [162–164].

#### Cross-linking with radiation techniques

According to the type of target tissues and polymer composition, photo-biomodulation can be done using a wide range of electromagnetic waves to induce covalent linkages within the polymeric network [165, 166]. The elimination of toxic residuals and eco-friendly procedures are associated with the application of irradiation for the fabrication of hydrogels [167, 168]. Microwave radiation can be easily applied in thermo-resistant polymers [169, 170]. The sensitivity of cells, drugs, and growth factors limits the bulk application of irradiation as chemical linkers for the preparation of hydrogels [171–173]. Although UV radiation can yield desirable outcomes for prompt gelation approaches the ionizing entity of UV can contribute to genotoxic effects such as DNA damage and mutation due to inappropriate repair, leading to reduced cell survival rate and the alteration of ECM components [174–176]. Besides these effects, juxtaposed tissues to the irradiated regions are closely exposed to increasing temperature values [177]. These features are less effective in consolidated tissues such as bone, and cartilage compared to the soft tissues [178, 179]. Therefore, attention should be taken to carefully regulate irradiation dose and reduce side effects on the surrounding niche while hydrogel mechanical properties remain intact [180]. Non-ionizing radiation such as visible light radiation is touted as an alternative approach [181]. This approach not only eliminates biosafety concerns related to UV radiation but also exhibits a deeper penetration rate. Thus, covalent linkages can be generated in deeper layers of hydrogels, increasing the gelation rate [182].

#### Enzyme-based hydrogel synthesis

The enzymatic cross-linking method has been at the center of attention in the fabrication of engineered hydrogels for skeletal muscle regeneration [183]. High-rate functionality of enzymes in physiological conditions and specific biocatalyst activity on certain substrates can reduce the side effects of applied substrates [184]. In general, the selection of enzymes for chemical cross-linking is based on the recognition of a specific substrate involved in the progression of a polymeric network [185, 186]. Among different enzymes, tyrosinase is a copper-based enzyme that generates covalent linkages via the oxidation of phenolic groups of tyrosine following the production of quinones [187, 188]. Large amounts of tyrosinase exist in plants and animal cells to append phenolic groups to the polymeric network [189]. However, the low specificity and non-toxicity of products make this enzyme a common enzyme type for the fabrication of hydrogels in the era of tissue engineering [189, 190].

Transglutaminases are other enzymes that are involved in cell adhesion, apoptosis, clotting, and signal transduction pathways [191, 192]. With an acyl transfer reaction between amines and γ-carboxamides, these enzymes can promote the formation of covalent bridges [193]. Unlike mammalian and plant transglutaminases, the activity of microbial transglutaminases depends on ionic calcium, resulting in an extensive application for tissue engineering studies [194, 195]. The high-rate specificity and selectivity limit the applicability of these enzymes for varied substrate types [196]. Peroxidases are other enzymes that form covalent bonds via the oxidation of phenol groups [197]. Properties like rapid gelation time, flexibility, and low specificity are advantages of these enzymes [198]. Among several peroxidase types, horseradish peroxidase (HRP) isolated from *Armoracia rusticana* has been extensively employed in the fabrication of hydrogels. HRP can be isolated from renewable sources with affordable costs for extraction and purification processes [199, 200]. However, the activity of HRP in the presence of hydrogen peroxide can yield reduced cell viability [201]. The indirect introduction of hydrogen peroxide via appending materials such as glucose oxidase (GOx) and D-glucose is an alternative strategy to circumvent the harmful effects of hydrogen peroxide [202, 203]. Sortase is prokaryote cysteine transpeptidase that forms covalent links between glycine and threonine in the LPXTG sequence. It has been shown that the activity of this enzyme is associated with cell attachment and pili synthesis in gram-positive bacteria. Among variant families of Sortase, Sortase A has been more utilized in tissue engineering applications due to its peculiar specificity and high-rate chemical reaction [204, 205].

#### Click chemistry modification

**Fig. 1:**
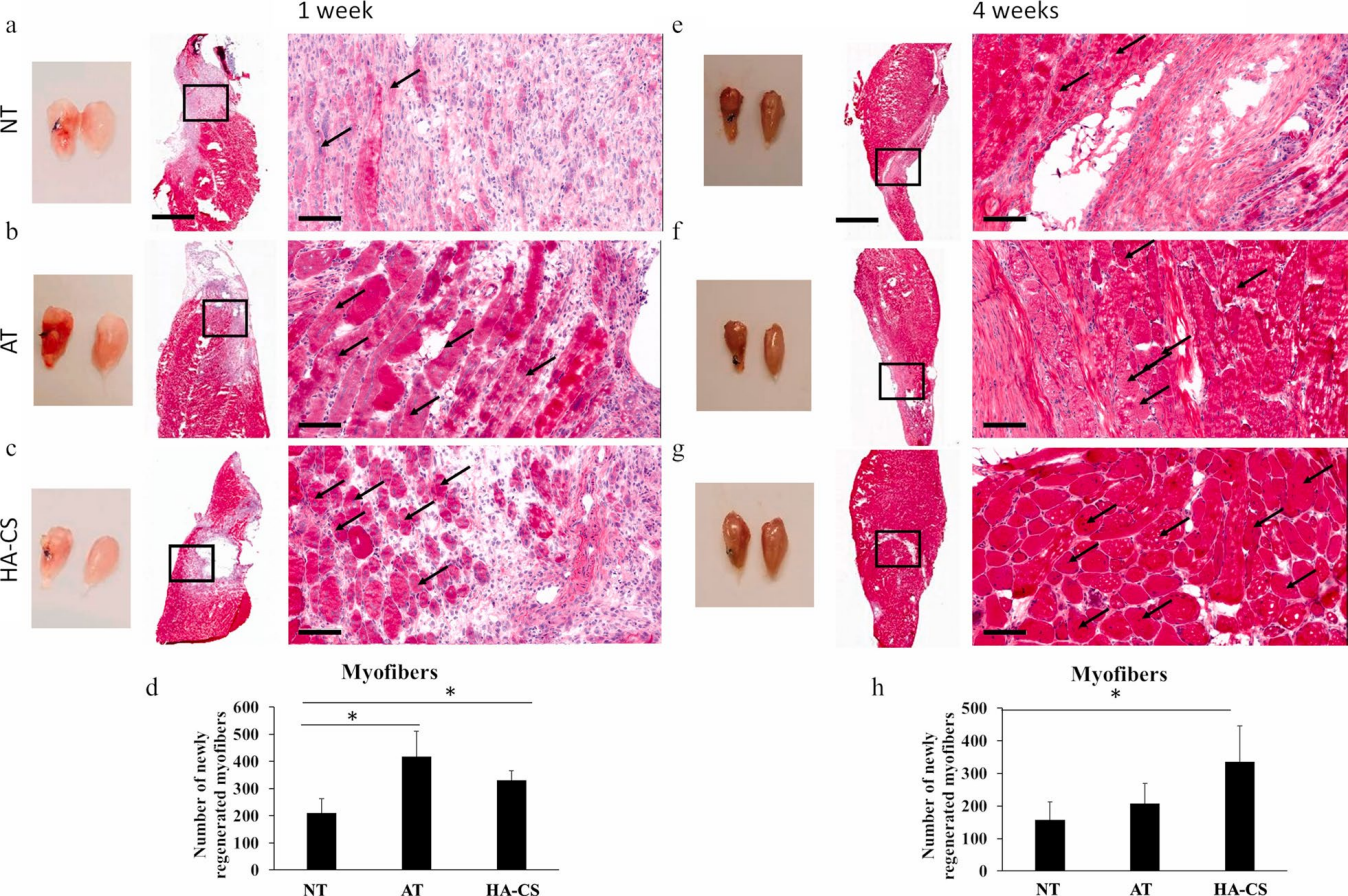
Gross appearance and histological examination of injured quadriceps muscles after transplantation of chondroitin sulfate-based HA hydrogel (HA-CS) compared to autograft treatment (AT) and no treatment groups (NT) (**a**-**h**). Macroscopic data indicate the volume of injury site is at the maximum size in NT mice compared to HA-CS and AT groups after 7 and 28 days post-transplantation (**a**-**g;** Scale bar: 2 mm). H & E staining confirmed the presence of a low number of newly generated fibers in NT as compared to AT and HA-CS groups (black arrows) after 7 days (**a**-**d:** Scale bar: 100 µm). Despite the reduction of defect size in all groups after 28 days, gross view revealed the prominent injured site in NT related to AT and HA-CS groups (**e–g**: Scale bar: 2 mm). The number of fibers reached maximum levels in HA-CS groups compared to NT mice. These values did not yield statistically significant differences between NT and AT groups (h; Scale bar: 100 µm). One-way ANOVA analysis with Tukey test; *p < 0.05. Adapted from [218]. (2021; Bioactive Materials; https://doi.org/10.1016/j.bioactmat.2020.10.012)

Synthesizing an ideal hydrogel with coordinated physical and biochemical behaviors can be performed using the click chemistry approach [206]. In hydrogels fabricated using click chemistry, the existence of unique links (known also as dynamic covalent bonds) makes these hydrogels easily mimic ECM-like behavior [207]. Flexibility, self-healing activity, and stability are the most prominent features in hydrogels fabricated using the click chemistry method. From the molecular aspect, various reactions between functional groups can lead to click chemistry reactions [208–210]. Each reaction technique per se possesses some advantages and disadvantages (Table 5). To date, the importance of ECM on cell activity and behavior has been demonstrated through numerous types of research [211–214]. Click chemistry provides modalities to investigate reciprocal cell-to-ECM interactions via the alteration of specific factors in a precise manner [215, 216]. Basurto et al. successfully synthesized flexible hyaluronic acid–base hydrogel using a thiol-ene reaction with a similar stiffness to skeletal muscle tissue. Short-term and long-term implantation of hyaluronic acid–base hydrogel in animals with volumetric muscle loss led to appropriate regenerative outcomes and mitigated inflammatory response [217]. In a similar study, the encapsulation of C2C12 myoblasts in the hyaluronic acid-based hydrogel with modulus similar to skeletal muscle tissue increased innervation, vascularization, and functional restoration of muscle tissue in the volumetric muscle loss animal model (Fig. 1) [218].

**Table 5:** Click chemistry for the fabrication of hydrogels

Type of click reaction	Functional group	Advantages	Disadvantages	Refs.
Azide-Alkyne reaction	Azide + alkyne	Bio-orthogonal injectable	High-temperature process Prolong gelation process	[326]
Diels–Alder reaction	Diel + Alkyne	No catalyst necessity Stereoselectivity	Prolong gelation process Non-injectable	[208]
Thiol-ene reaction	Thiol + alkene	Fast gelation process Oxygen and water resistance	Radical formation Accelerator requirements	[327]
Schiff base reaction	Amine + aldehyde	No cross-linker necessity Injectability	The unwanted reaction between aldehyde and amine groups in other molecules	[328]

Developing de novo methods for the delivery of cellular components to target tissues is one of the most interesting outcomes of the click chemistry system. In this method, cell surfaces and polymers with specific functional groups are modified to generate a cytocompatible covalent linkage between cells and the polymeric network [219, 220]. Using click chemistry, it is possible to increase cell adhesion properties and circumvent several limitations associated with peptide-related engineering approaches such as random protein folding during the absorption/appending phase and instability of covered surfaces [219]. In one study, the modification of alginate hydrogel with azide and alkyne sequences was done to make a covalent association between encapsulated C2C12 myoblasts and a polymeric network. Data revealed higher cell viability, and myogenic properties compared to classic cell loading method hydrogels [220].

## Chemical cues in skeletal muscle regeneration

The harmony of interactions/bio-interfaces between components of ECM and cells relies on chemical cues that provide steering signaling and cell adhesion sites, resulting in adequate cell-ECM responses to internal or external insulting factors [221–223]. Thus, hydrogel modification is essential with chemical agents such as signaling and adhesion factors to regulate cell viability, bioactivity, and regeneration processes [224, 225]. In particular, the existence of specific amino acid sequences with adhesion characteristics in the structure of several ECM proteins and glycoproteins known as cell-adhesion peptides (CAPs) is critical [226, 227]. Among different CAPs, arginylglycylaspartic acid (RGD) was first detected in the structure of fibronectin [228]. Studies related to the detection and function of ligand peptide sequences in ECM components have enhanced peptide science in tissue engineering strategies [224, 227, 229, 230]. Based on this information, tissue engineering strategies have involved the selection of suitable ECM components with specific CAPS for hydrogel synthesis [231], isolation of certain peptides from predesignated sources, or fabrication of peptides using peptide synthesizers (Biotage®, CEM®, LABX®) and subsequent hydrogel/scaffold modification are prevalent approaches for CAPs utilization in tissue engineering strategies [161, 232]. From the molecular structure, sequences such as DEGA, IKVAV, and PHSRN with cell adherence characteristics are present in the structure of collagen [233], laminin [234], and fibronectin [235], respectively. The biological properties of CAPS include promoting interactions with the surrounding environment and enhancing cell viability [236, 237], cell migration [238], proliferation [239], apoptosis [240], and differentiation [241] which is accomplished through the coupling of peptide domains to cell surface integrins that result in signal transition. Data have indicated the critical role of RGD-integrin interaction on cell fate, proliferation, and morphological adaptation on different therapeutic platforms used for skeletal muscle tissue engineering [242, 243]. Hence, the utilization of RGD sequence as a default cell adhesive factor became prevalent in many studies [244–248]. The features are associated with the high affinity of RGD to about 8 various receptors [249]. Campiglio and collogues used pectin along with RGD with C2C12 myoblasts to heal injured muscle tissue [70]. Pectin-RGD hydrogels were synthesized through the solvation of pectin in MES buffer solution and modification with RGD. Data indicated that the culture of C2C12 cells on electrospun pectin-RGD nanofibers promoted proliferation and differentiation after 7 days compared to the non-modified pectin group [70]. In an experiment, the dynamic growth of fibroblasts and murine satellite cells was studied on hyaluronic acid hydrogel modified with RGD, IKVAV, or VFDNFVLK sequences [250]. Data indicated that 2% hyaluronic acid‒RGD yielded the highest proliferation rate while IKVAV-modified hyaluronic acid substrate increased morphological adaptation and motility of plated cells. Interestingly, data showed that fibroblast migration is affected by peptide chains [250]. Due to the induction of myogenesis via the expression of Pax7 and MyoD, it was postulated that 3% hyaluronic acid with IKVAV sequence is an ideal substrate for the regeneration of muscle tissue. Previously, it was shown that RGD is an appropriate amino acid sequence to trigger the attachment of MSCs under hypoxic conditions [251]. Simultaneous application of RGD and IKVAV in specific concentrations enhances BMMSCs adhesion and morphological adaptation under different culture systems [252]. It is believed that environmental factors exert an inevitable role in RGD functionality via the direct alteration of peptide sequences [252]. For instance, data indicated increased human ECs attachment and proliferation rate after being plated on ELR substrate modified with bicyclic RGD peptides via direct interaction with integrins αvβ3 and α5β1 [252]. Blending various contents of RGD and IKVAV peptides in polystyrene-b-poly(ethylene oxide) base film led to the formation of structured actin fibers along with contractile actomyosin bundles in human MSCs. By increasing IKVAV levels, MSCs acquired round shape morphology with amorphous actin fibers at their peripheries [94]. Challenges and limitations can restrict the application of CAPs in tissue engineering strategies. For example, CAPs are expensive and isolated via using time-consuming procedures. In addition, most CAPs are sensitive to enzymatic digestion and their activities are reduced during the isolation procedures [253, 254].

## Induction of angiogenesis using scaffolds for muscle regeneration

The presence of vascular networks is essential for the functionality of all body organs [255]. These tubular structures act as platforms for microcirculation between blood and ECM to maintain cell homeostasis [256]. Without the participation of blood vessels and capillary networks, the durability of neo-regenerated tissues is not possible [257]. Hence, stimulation of an angiogenic response is parallel to functional tissue regeneration and efficient regenerative outcomes [102]. One reason that causes muscular mass atresia and injury is the lack of a suitable supporting vasculature system [258]. The term angiogenesis is defined as the formation of de novo vessels from the preexisting network in response to several signaling molecules during physiological and pathological conditions [259]. Regarding the fact that blood vessels guarantee suitable blood and oxygen resource throughout the tissues, using biomaterials with angiogenic potential are at the center of debate [259]. It was suggested that genetically modified cell-based tissue regeneration approaches can promote angiogenesis by the production of varied growth factors. The attachment of these factors to their cognate receptors on the EC surface increases neovascularization [260]. ECs furnish the luminal surface of the vascular system and can promote vascularization in response to pro-angiogenic factors [261]. It has been shown that pro-angiogenic factors such as VEGF, FGF, HGF, and HIF-1α mainly participate in vascular growth and expansion. These factors are released from different stem cell types such as EPCs, MSCs, and peripheral blood mononuclear cells and have been typically used for angiogenesis evaluations in several preclinical and clinical studies [262]. In response to the gradients of angiogenic factors, EPCs, MSCs, and peripheral blood mononuclear cells are recruited from different tissues, especially bone marrow, and accommodated the injured sites [263].

Hydrogel encapsulation methods with optimized procedure parameters have emerged as encouraging approaches to overcome cell leakage after injection into the injured muscles. It is possible to fabricate a safeguarded platform with proper physiochemical performance for transplanted cells to trigger the angiogenesis potential [264]. To achieve significant therapeutic effects with accurate fluid flow control, several hydrogel structures such as microgels, fibers, vascularized architectures, and perfusable single vessels have been generated from electrostatic droplet extrusion, micromolding, microfluidics, and 3D printing technologies, targeting the improvement in limb angiogenesis [265]. Besides, chemically modified hydrogels have been used in limb angiogenesis strategies benefiting from the regulation of cell-ECM interaction [266]. Along with other growth factors, IGF-1 has been shown to accelerate skeletal muscle renewal and inhibit cell apoptosis [267]. This factor can also increase stem cell immobilization and neovascularization via the activation intracellular PI3K signaling axis [268, 269]. The C terminus of IGF-1 (IGF-1C) with functional bioactivity can be linked to the scaffold structure for the regulation of angiogenic properties of encapsulated cells [270]. An artificial matrix including chitosan and hyaluronic acid modified by IGF-1C peptide was used to regulate the therapeutic neovascularization of AD-MSCs in ischemic limbs [271]. The transplantation of AD-MSC-load hydrogel enriched with IGF-1C led to improved blood perfusion and myogenesis via the secretion of pro-angiogenic factor angiopoietin-1 and regulation of immune cell infiltrate. Along with these changes, excessive collagen fiber deposition was reduced after the transplantation of hydrogel to the target sites [271]. In a study (phase I–IIa), gelatin microspheres were transplanted as a therapeutic angiogenesis system to 10 patients with CLI [272]. To this end, patients [with arteriosclerosis obliterans (*n* = 7) or thromboangiitis obliterans (n = 3)] received a 200-μg intramuscular injection of bFGF/gelatin hydrogel microspheres. Based on the obtained data, transcutaneous oxygen pressure was meaningfully improved in both subgroups 4 and 24 weeks after treatment, indicating the angiogenesis potential of gelatin hydrogel with sustained bFGF release into the muscular mass [272].

Elastin is an essential constituent of ECM and is promised biomaterial in skeletal muscle and vessel reconstitution because of its appropriate mechanical strength and elasticity [273]. Elastin plays an important role in the stimulation of cell signaling cascades associated with proliferation and angiogenesis [274]. Elastin-like recombinamers (ELRs) are bio-engineered polypeptides with pentapeptide (VPGXG) repeat, where X can be any amino acid excluding proline [275].

**Fig. 2:**
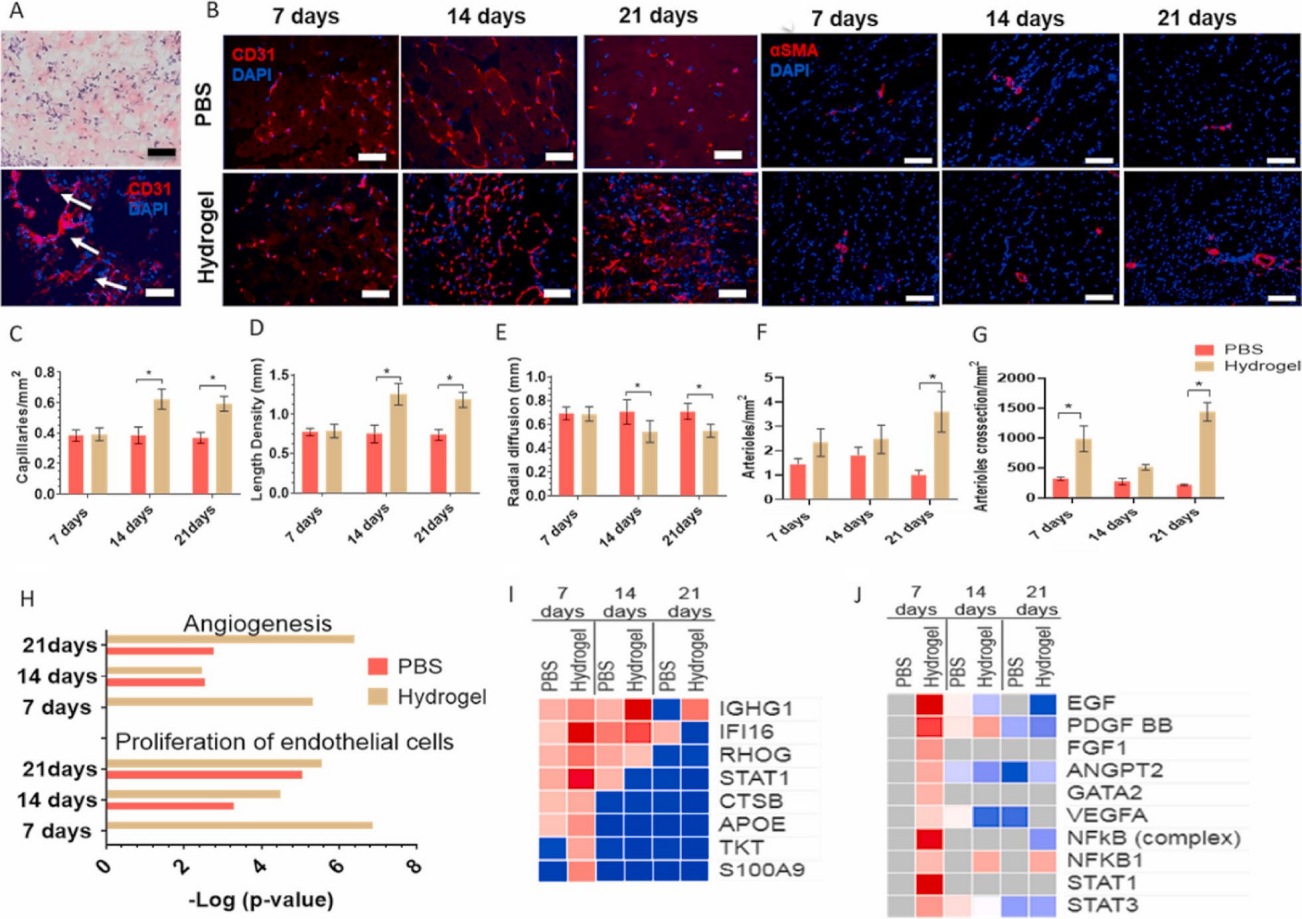
Angiogenesis properties of ELR-based hydrogel transplanted in a mouse model of limb ischemia (**A–J**). Bright-field imaging indicates the formation of de novo vessels within the ELR-based hydrogel on day 7. CD31^+^ vessels are indicated using immunofluorescence staining (**A**; Scale bar: 100 µm). Immunofluorescence staining revealed the formation of α-SMA^+^ arterioles and CD31^+^ vessels in ELR-treated mice compared to the PBS group (**B**; Scale bar: 20 µm). Mean capillary density (**C**; Scale bar: 20 µm); mean length density (**D**; Scale bar: 20 µm); stereological quantification (**E**; Scale bar: 20 µm); arteriole density (**F**; Scale bar: 20 µm); and mean the number of cross-sectioned arterioles (**G**; Scale bar: 20 µm). Proteomic analysis of ELR-treated group related to PBS mice (**H**-**J**). Heat map analysis of angiogenesis factors (**I**) and their relative upstream factors (**J**). *n* = 6; Two-way ANOVA analysis.*p < 0.05. Adapted from [273]. (2021; Biomaterials; https://doi.org/10.1016/j.biomaterials.2020.120641)

ELRs benefit from characteristics such as elasticity, low thrombogenicity, self-assembly, and thermoresponsive behavior, comparable to those of natural elastin but compensate for elastin insolubility and restricted obtainability of sources (Fig. 2) [276]. Recently, in vitro and in vivo angiogenesis properties of ELRs have been reported [274]. ELRs have also been fabricated via the click chemistry method with different sequences such as HE5-cyclooctyne carrying the matrix metalloproteinases (MMPs) binding site and HRGD-azide with an RGD sequence. ELR-based hydrogel endorsed the formation of new arteries, ECM remodeling, glycosylation, and protein signaling cascades in several tissues [277]. Based on several reports, polymers including multidomain nano-peptides may stimulate angiogenesis and muscular restoration during muscle loss injuries [278]. As an instance, neuropeptide-Y (NPY3-36)-loaded copolyoxalate containing vanillyl alcohol (PVAX) showed promised angiogenic effect in ischemia-induced adult C57BL/J6 mice with simultaneous reduction of infarction size and mortality rate [279]. Furthermore, 3D printing techniques including inkjet, layer-by-layer and thermal extrusion, stereo-lithography or digital light processing, and photo-degradation can be used to control the three-dimensional organization and distribution of bio-gels [280]. More investigations are needed to find adequate hydrogel types with specific physicochemical properties and spatial organization for the acceleration of angiogenesis in injured tissues.

### Induction of angiogenesis and myogenesis via exosome-loaded hydrogels

Exosomes (Exos), a subclass of extracellular vesicles, have a crucial role in paracrine cell-to-cell interaction via the transfer of several signaling biomolecules [280, 281]. These particles with an average diameter size of 50–150 nm can promote angiogenesis in ischemic organs [282]. It has been shown that Exos lack oncogenic and immunogenic features and can cross biological interfaces and thus distribute easily in the whole body [283]. Due to the existence of specific myogenic factors like IGFs, bFGF, EGF, HGF, etc. Exos are thought to be involved in the regeneration of injured muscle tissue [280]. Besides, the exosomal amino acids from different sources can promote myogenesis via the regulation of protein synthesis and basal metabolism. In an experiment conducted by Mobley and co-workers, incubation of C2C12 myoblasts with whey-derived Exos improved myotube diameters and length via the modulation of eIF4A due to higher l-leucine contents [284]. Of note, miRNAs with specific properties participate in myocyte function via the regulation of protein-coding mRNAs [285]. The existence of particular genetic elements makes Exos a therapeutic candidate for the treatment of pathological conditions and the alleviation of congenital disorders. It was indicated that the injection of bone marrow MSC-derived Exos blunted the reduction of myotube diameter induced by dexamethasone in C2C12 myoblasts by the upregulation of miR-486-5p and down-regulation of FoxO1. Along with these data, MSC Exos reduced muscle atrophy via the modulation of the miR486-5p/Foxo1 axis in a mouse model of muscle atrophy [286].

**Fig. 3:**
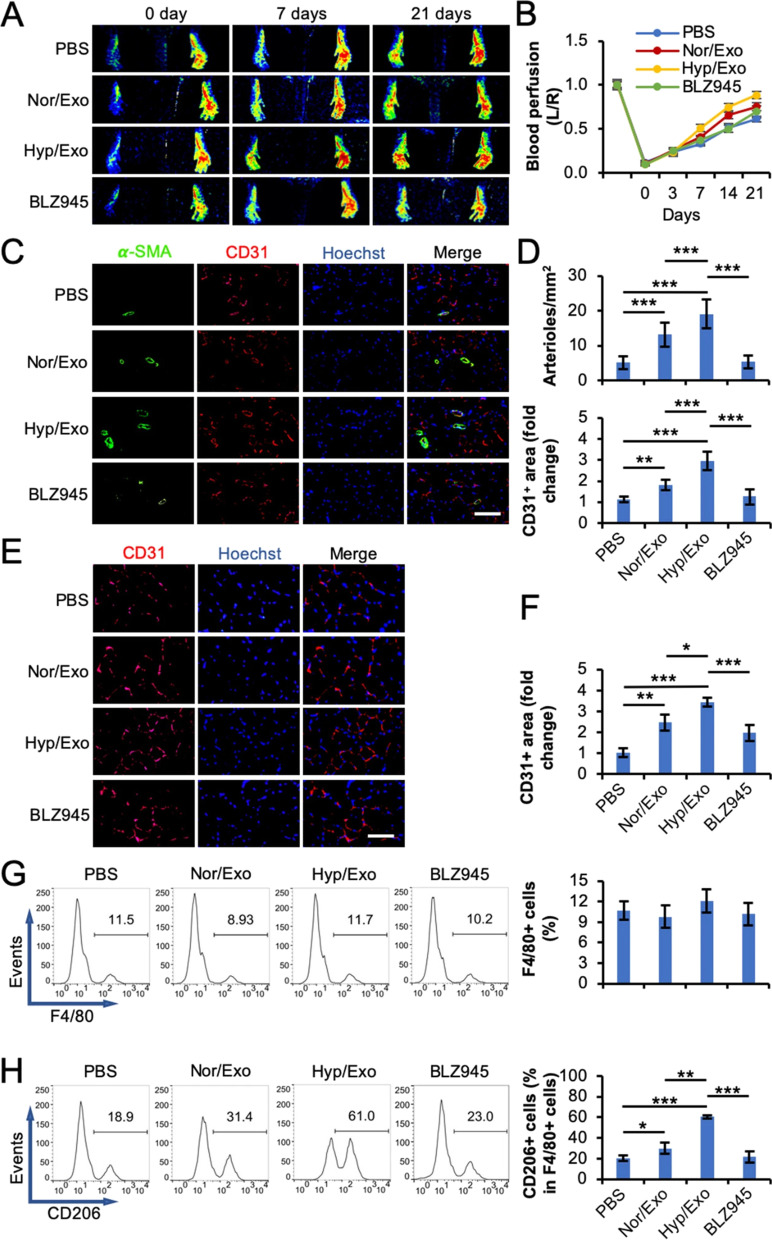
Evaluation of angiogenic and immunomodulatory properties of ASC-derived Exos in a mouse model of ischemic muscle injury (**A**-**H**). Ischemic muscle injury was induced by the ligation of the femoral artery. Mice were allocated into PBS; Normal Exos (Nor/Exo); Hypoxic Exos (Hyp/Exo); and Hyp/Exo + BLZ945 groups (each in 8). Laser speckle imaging indicated the changes in hind paw blood perfusion after 21 days (**A**). Analyses confirmed superior effects of Nor/Exo, especially Hyp/Exo, on the promotion of plantar perfusion (**B**). Immunofluorescence imaging of injured adductor muscles 3 weeks after injection of Exos (**C** and **D**). Data indicate the promotion of α-SMA^+^ and CD31^+^ vessels in mice that received ASC hypoxic and normoxic Exos compared to the PBS group (Scale bar: 150 µm). Simultaneous injection of BLZ945 blunted these effects (Counterstaining: Hoechst 33,342). Immunofluorescence imaging of CD31 in injured gastrocnemius muscles after injection of Exos (**E** and **F**; Scale bar: 100 µm). An average number of CD31^+^ vessels increased following the injection of hypoxic and normoxic Exos compared to the PBS mice. Again, BLZ945 blunted these effects. Flow cytometry analysis of F4/80 + macrophages (**G**) and M2 type CD206^+^ macrophages (**H**). One-Way ANOVA analysis with Tukey method. *p < 0.05, **p < 0.01, and ***p < 0.001. Adapted from [287]. (Copyright 2020; Stem Cell Research & Therapy; https://doi.org/10.1186%2Fs13287-020-01669-9)

Emerging data have shown that environmental factors such as oxygen levels can affect the angiogenic properties of stem cell Exos [287]. Zhu and colleagues investigated the angiogenic and immunomodulatory properties of adipose-derived stem cell (ASC) Exos under normoxic and hypoxic conditions in a mouse model of the ischemic hind limb (Fig. 3) [287]. Data indicated that ASC Exos can promote M2-type macrophage polarization (CD206^+^ cells), and suppress CD86 macrophages. Along with these changes, increased α-SMA^+^ and CD31^+^ vessels were obtained in ischemic muscle after injection of hypoxic and normoxic Exos [287]. Despite the existence of therapeutic properties, several obstacles limit the application of Exos in the clinical setting [261]. For example, direct transplantation causes short-time Exo stability because of mechanical stress and activation of immuno-reactive phagocytes [261]. Co-transplantation of Exos with supporting substrates yielded promising outcomes in different experiments. Integration of human placenta MSC Exos with chitosan hydrogel increased the stability of exosomal miRNAs, and proteins in in vivo conditions, leading to improved myogenesis and angiogenesis in a mouse model of hindlimb ischemia [288]. In an experiment performed by Rolland and co-workers, the treatment of muscle progenitor cells with NF-κB and PD-L1 enriched platelet Exos increased proliferation rate and differentiation capacity [289]. Injection of Exo-loaded collagen + fibrin glue hydrogel in a rodent model of muscle injury contributed to the healing of *latissimus dorsi* via the polarization of macrophage toward M2 type [289].

## Local immunomodulation in muscular tissue using hydrogels

Over the past decades, the application of several agents to modulate the host immune system has had a major impact under pathological conditions [290, 291]. In short, an inflammatory response occurs due to the activation of different cell types such as mast cells, macrophages (M1/M2), monocytes, lymphocytes, neutrophils, and dendritic cells. As a correlate, the production of cytokines, ROS, and infiltration of immune cells lead to the destruction of target tissues [292]. To mitigate these conditions, immunomodulation approaches are at the center of attention [293]. Recent studies have therefore focused much attention on the area of local immunomodulation through various immunomodulatory nanosystems (IMNs) [291]. In general, IMNs have been developed from engineered NPs, small drugs, nanomaterials, and biomaterials, factors such as cytokines, antibodies, siRNA, extracellular vesicles, and polysaccharides [100, 292, 293]. It was suggested that scaffolds provide a platform for immune cell adhesion, proliferation, and differentiation. Based on engineered shape, geometry, topography, pore size, physiochemical properties, and surface units, the local immunomodulatory properties of scaffolds can be regulated [294]. For instance, Tylek and co-workers developed a box-shaped PCL fibrous scaffold with inter-fiber spaces ranging from 40 to 100 µm. The culture of freshly isolated human monocytes led to cell elongation and orientation toward M2 macrophage phenotype. These effects were prominent in scaffolds with smaller sizes near 40 µm [294]. However, further researches are mandatory to determine the appropriate scaffolds with certain dimension and surface decoration for the modulation of immune cell reaction and uncontrolled fibrosis [293, 295, 296]. In another study, the critical role of other parameters such as pore size and scaffold composition was determined in terms of mast cell activity [297]. The mutual interaction of mast cells and scaffold was decreased by increasing the levels of polydioxanone, leading to a reduction of IL-6 and TNF-α, and induction of VEGF. Along with these data, it was suggested that pore sizes more than 4 µm blunted the activation of mast cells in in vitro settings [297]. Besides, diverse immunomodulatory agents like drugs, NPs, proteins, cytokines, and anti-ROS composites can be added to transplant scaffolds to control unwanted immune responses [292]. Likewise, co-transplantation of scaffolds and certain cell types, *i*.*e*. MSCs, with immunomodulatory properties is also helpful [293]. It is thought that the simultaneous induction of anti-inflammatory factors along with the suppression of pro-inflammatory cytokines is another strategy during the transplantation of scaffolds or hydrogels into the injured muscular tissue [298]. In this regard, Shortridge and colleagues investigated the anti-inflammatory properties of genipine cross-linked injectable PCL/collagen hydrogel after exposure to digestive inflamed sites [298]. They cross-linked IL-4 conjugated PCL nanofibers with type I collagen using genipine. This procedure led to prominent stability of the hydrogel polymeric network and reduced release of IL-4. The incorporation of IL-4 into the PCL/collagen backbone inhibited the local production of TNF-α, and COX-2 and increase macrophage polarization toward the M2 type [298]. The culture of mouse C2C12 myoblasts on cross-linked hydrogel with 1% genipine led to a reduction in survival rate compared to 0.5% genipine and genuine-free hydrogel. One reason would be the reduction of porosity and increase in mean fiber diameter. Despite the promising anti-inflammatory effects of genipine on some cytokines, it is thought that genipine regulatory action is associated with microenvironment pH values [298]. As mentioned above, injectable ROS-scavenging hydrogels are promising approaches for the regeneration of injured muscular tissue. Shan and co-workers used mouse Luc^+^/GFP^+^ MSCs loaded within the ROS-scavenging hydrogel for the regeneration of ischemic muscles [299]. In the presence of hydrogen peroxide, MSCs proliferated via the activation of the PI3K/Akt/mTOR signaling axis when encapsulated within the ROS-scavenging hydrogel. The exposure of encapsulated MSCs inside the hydrogel to LPS-activated RAW264.7 macrophages led to the suppression of CD80^+^ cells and an increase in macrophages with CD206 surface markers in in vitro conditions [299]. As expected, this strategy led to the reduction of IL-1β, -6, and TNF-α and the increase in IL-4, and CD206^+^ macrophages after transplantation into the injured muscle mass [299]. In an interesting experiment, Lee et al. synthesized injectable hydrogel consisting of CD146, IGF-1, type I and III collagen, and poloxamer 407 for muscle tissue engineering. They believed that the attachment of CD146 to surface VEGFR-2 can stimulate efferocytosis in neutrophils and macrophages, leading to the reduction of pro-inflammatory cytokines while the simultaneous release of IGF-1 promotes the regeneration of muscles via the differentiation of muscle progenitor cells [300]. They found that the injection of hydrogel in the mouse model led to the activation of autophagy via the induction of ATG5, ATG7, LC3BII, Beclin-1, and P62. Along with these changes, the levels of factors associated with myogenesis such as Myogenin, eMyHC, MyCHII, and AERG increased which coincided with the reduction of inflammatory cytokines like NF-κB and IKβ [300]. Histological examination indicated the existence of efferocytosis and accumulation of CD11b/CD206 macrophages. These features increased the phagocytosis of injured myocytes at early stages after hydrogel injection, resulting in an accelerated healing process [300]. The in situ inhibition of immune cells is touted as another promising strategy to cease inflammatory response at the site of injury using injectable hydrogels [301]. In a study conducted by Alvarado-Velez and co-workers, they synthesized immuno-suppressive agarose hydrogel containing Fas ligand to initiate apoptosis in CD8^+^ lymphocytes and increase the therapeutic activity of transplant MSCs at the site of spinal cord injury [301]. Data indicated that Agarose-FasL hydrogels had the potential to increase the viability of allogenic MSCs and reduce infiltration and the number of CD8^+^ lymphocytes to the site of injury via the activation of surface cell marker CD90 and apoptotic death [301].

## Conclusion

**Fig. 4:**
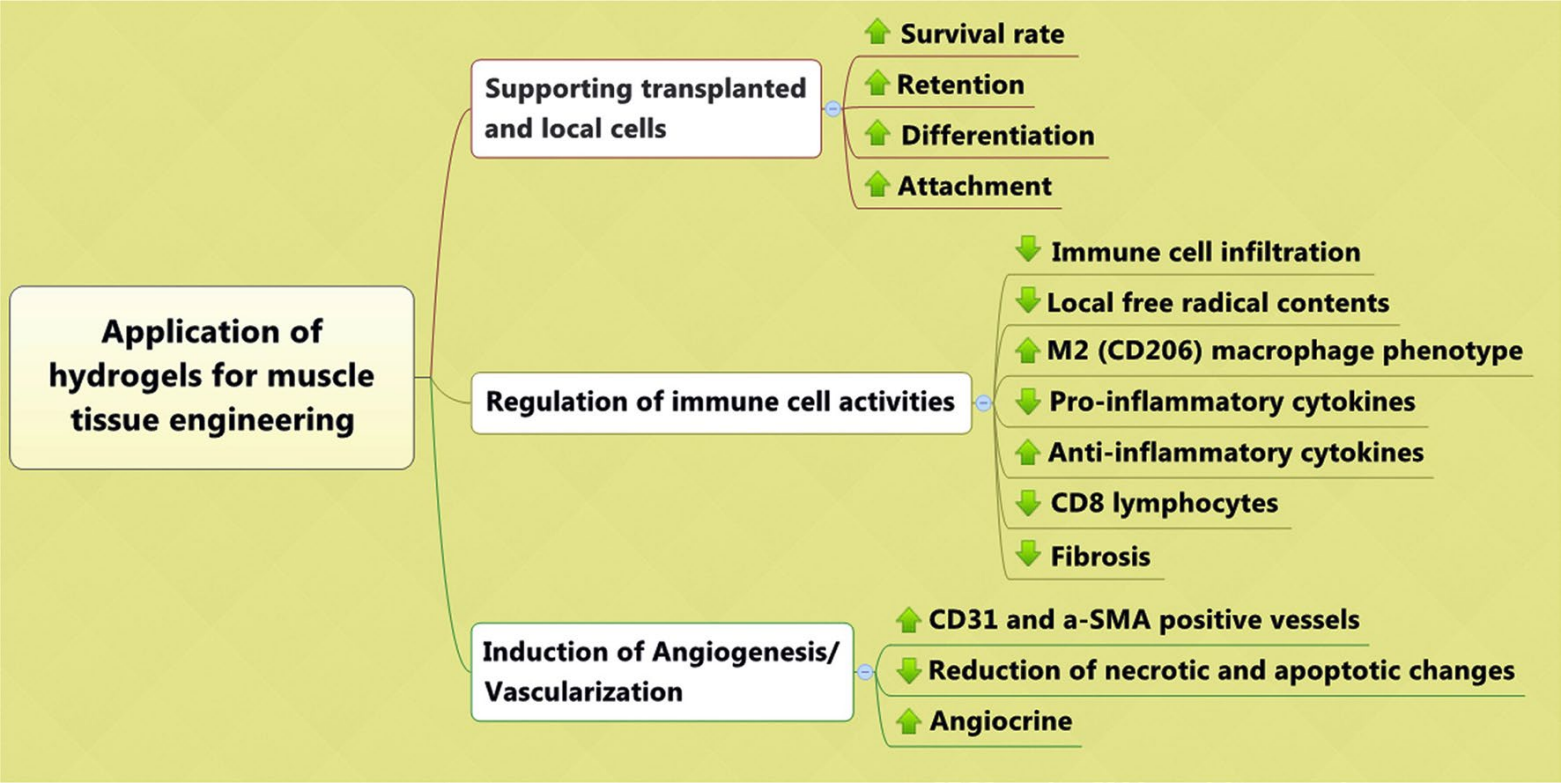
Several parameters should be considered for the regeneration of injured muscle tissue using hydrogels

**Fig. 5:**
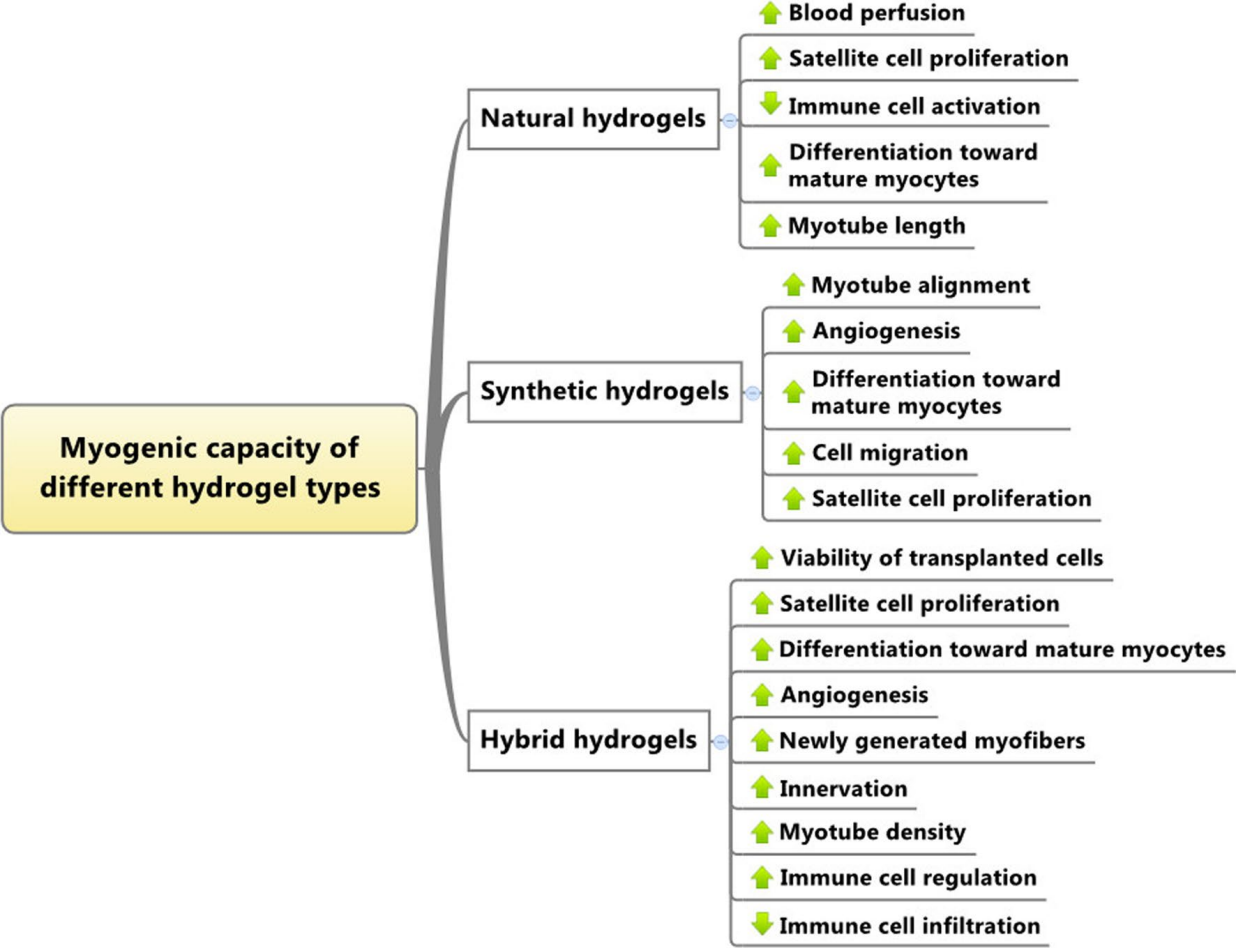
Proposed regenerative potential of varied hydrogel types in muscle tissue engineering

Fabrication and development of hydrogels with certain structures are mandatory to accelerate the regeneration of injured muscle tissue. Due to the unique structure of muscle tissue, applied hydrogels should possess certain physicochemical properties to activate the maturation of quiescent muscle progenitor cells toward mature myocytes. To achieve efficient muscle tissue regeneration, the regulation of angiogenesis and immune cell function will rely on using several hydrogel types (Fig. 4). The applied hydrogels should be engineered to support the survival and retention of transplanted cells after grafting into the injured cells. It should not be forgotten that the myogenic properties of varied hydrogel types are relatively different (Fig. 5). Despite recent advances in the preparation and synthesis of hydrogels for muscle tissue engineering, the application of sophisticated modalities like 3D and 4D printing approaches with novel engineering modalities will be helpful to attain better regenerative outcomes. With regards to the muscle tissue stiffness and physical properties, attempts should be focused on the finding most suitable substrates with proper mechanical features and concurrent myogenic capacity.

## Data Availability

Not applicable.

## Abbreviations

PAACA: 6-Acrylamidohexanoic acid
AD-MSCs: Adipose-derived mesenchymal stem cells
RGD: Arginyl-glycyl-aspartic acid
ASC: Adipose stem cells
BMMSCs: Bone marrow mesenchymal stem cells
CAPs: Cell adhesion peptides
CLI: Critical limb ischemia
COX-2: Cyclooxygenase-2
DTT: Dithiothreitol
EDGE: Ethylene glycol diglycidyl ether
ELRs: Elastin-like recombinamers
ECs: Endothelial cells
eNOS: Endothelial nitric oxide synthase
EPCs: Endothelial progenitor cells
EDGE: Ethylene glycol diglycidyl ether
Exos: Exosomes
ECM: Extracellular matrix
FGF: Fibroblast growth factor
GO: Graphene oxide
GOx: Glucose oxidase
HGF: Hepatocyte growth factor
HRP: Horse radish peroxidase
HIF-1α: Hypoxia-inducible factor-1α
IGF-1: Insulin-like growth factor 1
MMPs: Matrix metalloproteinases
NPs: Nanoparticles
PEGDA: Poly(ethylene glycol) diacrylate
PVA: Poly(vinyl alcohol)
PAA: Polyacrylic acid
PEO: Poly(ethylene oxide)
PLGA: Poly(lactic-co-glycolic acid)
PCL: Polycaprolactone
PEG: Polyethylene glycol
PLA: Polylactic acid
PPO: Polypropylene oxide
PU: Polyurethane
ROS: Reactive oxygen species
TNF-α: Tumor necrosis factor-α
VEGF: Vascular endothelial growth factor
VEGFR: Vascular endothelial growth factor receptor
